# Supplementary figures and images for: The Wolbachia WalE1 effector alters Drosophila endocytosis
Source: bioRxiv. 2023 Feb 27:2023.02.26.530160. Preprint. [Version 1] doi: 10.1101/2023.02.26.530160 (PMC10002650; doi:10.1101/2023.02.26.530160)

# Supplementary Figures:

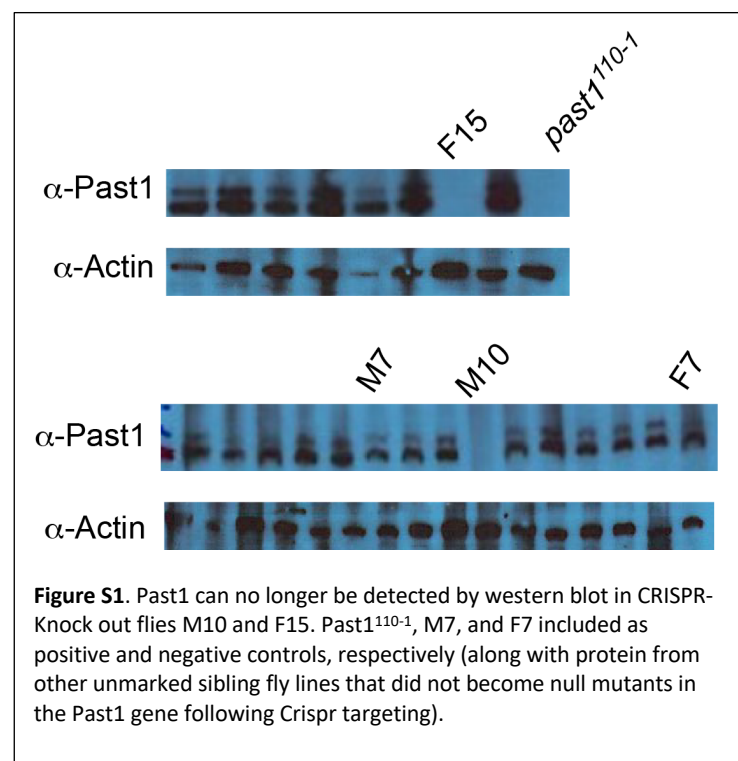

Supplement: Supplement 1 [file NIHPP2023.02.26.530160v1-supplement-1.pdf]
